# Nutritional Follow-Up in Indigenous Children Under Five Years in Colombia

**DOI:** 10.3390/children13070880

**Published:** 2026-06-30

**Authors:** Pedro Barrera-López, Andrés Felipe Mora-Salamanca, Kevin Rico, Sandra Barrera-Ayala

**Affiliations:** Grupo de Salud Materna y Perinatal, Instituto Nacional de Salud, Avenida Calle 26 No. 51-20-Zona 6 CAN, Bogota 111321, Colombia; pbarrera@ins.gov.co (P.B.-L.); afmorasa@unal.edu.co (A.F.M.-S.); krico@ins.gov.co (K.R.)

**Keywords:** child, indigenous peoples, infant nutrition disorders, child nutrition disorders, social determinants of health, Colombia

## Abstract

**Highlights:**

**What are the main findings?**

- Nutritional alterations were highly prevalent among Wayúu children under five years of age in Manaure, with chronic malnutrition and stunting being the dominant pattern, whereas acute malnutrition was comparatively infrequent.
- In the exploratory longitudinal follow-up subgroup, descriptive analysis of anthropometric indicators suggested possible patterns of growth impairment over time, particularly in weight-for-age and height-for-age indicators. Older children showed significantly lower values in these indicators compared with younger children, with no significant differences by sex.

**What are the implications of the main findings?**

- Nutritional surveillance in Indigenous preschool children should prioritize early detection of chronic growth impairment and sustained follow-up, not only screening for acute wasting.
- These findings support the need for culturally appropriate, multisectoral interventions that address food insecurity, access to health services, and the structural determinants that perpetuate malnutrition.

**Abstract:**

Background/Objectives: Indigenous children in La Guajira, Colombia, live in a context of structural vulnerability that may compromise growth and nutritional status. This study aimed to characterize anthropometric patterns and longitudinal nutritional changes in Wayúu children under five years of age. Methods: A quantitative cross-sectional analysis was conducted in 398 children from 27 Wayúu communities in Manaure, La Guajira, Colombia, with an exploratory longitudinal follow-up subgroup assessed over an 8-month period. Anthropometric measurements were obtained by trained pediatricians and classified using standard WHO growth references. Descriptive and bivariate analyses were performed for the full sample, and exploratory longitudinal changes were assessed in the follow-up subgroup. Results: At baseline, 92.46% of children presented at least one nutritional alteration, and 89.95% had malnutrition or nutritional impairment. Stunting was the most frequent condition (60.1%), whereas acute malnutrition was less common. In the exploratory longitudinal subgroup, 41.67% of children showed descriptive changes in at least one anthropometric indicator, with a significant increase in nutritional risk between visits. Older children showed significantly lower weight-for-age and height-for-age values than younger children, while no significant differences were observed by sex. Conclusions: Wayúu children under five years in Manaure show a pattern dominated by chronic growth impairment with worsening anthropometric indicators over time. These findings highlight the need for sustained, culturally adapted, and multisectoral strategies to prevent and manage childhood malnutrition in Indigenous populations.

## 1. Introduction

Malnutrition and its impact on early childhood development (ECD) are a priority for public health due to their effects on survival, neurodevelopment, future productivity, and social equity. Malnutrition and its implications extend beyond medical landscape, becoming a cross-cutting area of interest for societies and governments. Therefore, the Sustainable Development Goals emphasize the global commitment to combat malnutrition and reduce its impact on child development (Goal 2) [1,2].

ECD encompasses the period from gestation through the first 6–8 years of life. This stage is critical for the acquisition of physical, cognitive, emotional, and social skills that determine well-being across the life course, with implications at both individual and societal levels [3,4]. Hence this period is particularly sensitive to adverse exposures such as food insecurity, recurrent infections, and psychosocial stress.

To achieve optimal development, the World Health Organization (WHO) and UNICEF promoted the “nurturing care” concept, which integrates the following components: good health, adequate nutrition, safety and protection, responsive caregiving, and opportunities for early learning [4,5,6,7]. However, these elements alone are insufficient. A supportive social and economic environment, which may either enhance or constrain child development, is required [4,5,8,9]. The impact of these social determinants is substantial: worldwide, an estimated 200 million children under five fail to reach their developmental potential due to poverty, food insecurity, limited access to healthcare, and inadequate early stimulation [10].

In Colombia, several populations experience similar conditions of deprivation and social vulnerability. Among them are Indigenous communities, particularly the Wayúu population in La Guajira. This community faces structural challenges that increase the risk of malnutrition and developmental disorders, including persistent poverty, chronic food insecurity, lack of access to drinking water and basic sanitation, residence in geographically dispersed rural areas with limited accessibility, cultural and language barriers to healthcare access, low educational attainment, structural discrimination, and limited local institutional capacity [11,12,13,14]. Additionally, the Wayúu population is affected by climate change, subsistence-based economies, migration, and fragile social protection systems, all of which further compromise access to adequate nutrition. These conditions create a detrimental environment that directly impacts child nutrition and development, perpetuating an intergenerational cycle of inequality and poor health outcomes [3,8,11,15,16]. Nevertheless, despite the conditions described above, it remains necessary to characterize the nutritional status of this population and identify potential risk factors in order to establish targeted interventions aimed at combating childhood malnutrition in a particularly vulnerable group such as the Wayúu community.

Child development is intrinsically linked to nutritional status [4,6,17], which in turn varies according to socioeconomic and environmental conditions [3,8,14]. Consequently, it is essential to establish global reference standards defining optimal growth. The WHO Child Growth Standards represent a methodological milestone, derived from multicenter cohorts of healthy children raised under optimal conditions, describing how children should grow rather than how they grow in specific contexts [18,19]. These standards enable early detection of deviations and guide both clinical and population-level interventions, including in vulnerable populations such as the Wayúu, distinguishing between normal constitutional variation and growth alterations secondary to deprivation. They are critical tools for identifying nutritional risk, monitoring interventions, and evaluating recovery and developmental outcomes [10,18,20,21].

Given the impact of nutrition and social determinants on child development [8,17], and the vulnerability of populations such as the Wayúu [11,13], multiple governmental strategies have been implemented to reduce disparities. These include strengthening institutional presence, generating local data, implementing culturally sensitive primary care programs, integrating nutrition, health, education, and social protection, and promoting community empowerment to develop sustainable and culturally appropriate policies [2,22].

At the national level, the Colombian government established the Health Research Fund (FIS), administered by the Ministry of Science, Technology, and Innovation, to finance health research projects [23,24]. Through this mechanism, resources were allocated to the National Institute of Health (INS) to develop the project: “Community intervention for the prevention of maternal and perinatal morbidity and mortality under a sexual and reproductive health approach, and assessment of growth and development in Wayúu children in a region of La Guajira” (in Spanish, “*Intervención comunitaria para la prevención de la morbimortalidad materna y perinatal bajo el enfoque de salud sexual y reproductiva; y valoración del crecimiento y desarrollo en niños y niñas del pueblo Wayúu de una región de La Guajira*”). This project includes multiple phases, beginning with the characterization of population and nutritional status as a foundation for understanding structural vulnerabilities and guiding interventions.

Therefore, we aimed to identify growth and developmental patterns in a cohort of Wayúu children in La Guajira, Colombia, within a national governmental initiative to reduce child morbidity and mortality and strengthen ECD in vulnerable populations.

## 2. Materials and Methods

### 2.1. Study Design

This study is part of a government-led mixed-methods initiative incorporating a qualitative component based on a phenomenological and participatory framework, as well as a quantitative component initially designed as a prospective cohort study.

This article presents the results of a quantitative analysis of the cross-sectional assessment of anthropometric growth patterns among Wayúu children in La Guajira, including a subgroup followed longitudinally to evaluate growth patterns over an 8-month period (from 12 August 2024 to 28 March 2025).

### 2.2. Study Population and Sample

A community census was conducted including all children under five years of age from 27 target communities in the municipality of Manaure, La Guajira, Colombia. Communities were selected based on the willingness of local Indigenous authorities to participate in the intervention. A total of 398 children were included, representing:

• 0.41% of the total population in La Guajira within this age range [25,26,27];
• 3.38% of the population of the municipality of Manaure according to the 2018 census [26];
• 3.75% of children under 4 years in the dispersed rural population of Manaure;
• 3.51% of the total Indigenous population under 4 years [25,27,28].

**Figure 1:**
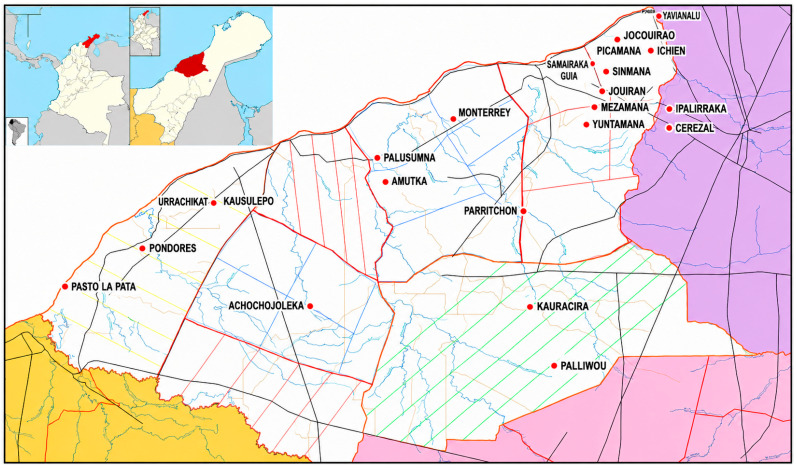
Geographical location of the municipality of Manaure, La Guajira, Colombia. The red outline indicates the location of La Guajira Department within Colombia and the municipality of Manaure within La Guajira. The numbered areas represent the communes within the municipality. Neighboring municipalities are highlighted as follows: Riohacha (yellow), Maicao (lilac), and Uribia (purple). Source: Authors’ own work.

All participants belong to the Wayúu ethnic group, in a region where 93.6% of the population self-identifies as Indigenous. The study area covers 1643 km^2^ with a population density of 45 inhabitants/km^2^ [29,30] (Figure 1). Despite the sample size and broad territorial coverage, the use of non-probabilistic sampling introduces selection bias; therefore, the findings may not be representative of the overall nutritional status of the Wayúu pediatric population.

### 2.3. Inclusion and Exclusion Criteria

All children under five years of age (0–4 years) residing in the selected communities at the time of the community intervention were included. No exclusion criteria or recruitment restrictions were applied. Participants were assessed in the presence of their legal guardians, who provided informed consent prior to data collection.

### 2.4. Variables and Data Collection

Data were collected by trained pediatricians using calibrated WHO/PAHO-recommended anthropometric equipment. Measurements were recorded in metric units, and uncertain assessments were verified by a second pediatrician. Information was initially documented in paper-based clinical records and later entered into a digital database. Collected variables included sociodemographic characteristics and anthropometric indicators classified according to WHO Anthro standards.

### 2.5. Statistical Analysis

Demographic and anthropometric variables classified according to WHO Anthro standards were analyzed descriptively. Bivariate analyses using chi-square and nonparametric tests were performed to compare anthropometric indicators by sex, and Spearman correlation analyses assessed the relationship between age and nutritional classification. Analyses were conducted with a 95% confidence interval, considering *p* < 0.05 as statistically significant. Growth patterns were additionally evaluated in the longitudinal follow-up subgroup. Data management was performed using Microsoft Excel, and statistical analyses were conducted with SPSS version 21.

## 3. Results

A total of 398 children were included in the study: 216 boys and 182 girls, corresponding to a male-to-female ratio of 1.16 and a proportion of 54.3% male participants (Table 1). The median age was 2.65 years (IQR: 1.47–3.57) among boys and 2.76 years (IQR: 1.48–3.77) among girls, consistent with the sex distribution reported in the most recent departmental census.

**Figure 2:**
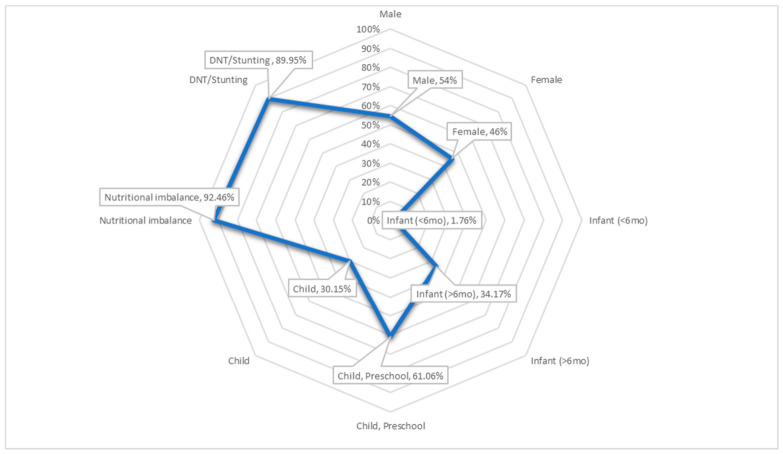
Distribution of sex, age and nutrition disorders among Wayúu Indigenous children under five years from Manaure, La Guajira, Colombia.

Overall, age distribution was non-normal, with a median of 2.70 years (IQR: 1.48–3.67; range: 3.25 months to 5.05 years). The majority of participants (98.2%) were older infants and children aged between 6 months and 5 years, while only 1.8% were younger infants. Age distribution was similar across sexes (Figure 2).

Follow-up was achieved in 6.03% of cases (*n* = 24), and 12 children changed communities during the study period.

### 3.1. Baseline Nutritional Status

At the initial evaluation, more than 90% of participants presented at least one form of nutritional alteration. Except for 10 cases, all children exhibited some degree of nutritional impairment or established malnutrition.

The most prevalent condition was stunting (height-for-age deficit), affecting 60.1% of participants, followed by underweight (weight-for-age deficit) at 23.1%. In contrast, the prevalence of acute malnutrition (wasting) was low, at 3.5% according to weight-for-height and 2% according to MUAC.

These findings suggest a pattern of chronic growth impairment, which also affected head circumference, with nearly half of the children (46.48%) classified as having microcephaly (Table 2).

Overall, 92.46% of participants had at least one nutritional alteration, and 89.95% had some form of malnutrition or nutritional impairment (Figure 2).

### 3.2. Exploratory Longitudinal Follow-Up

**Figure 3:**
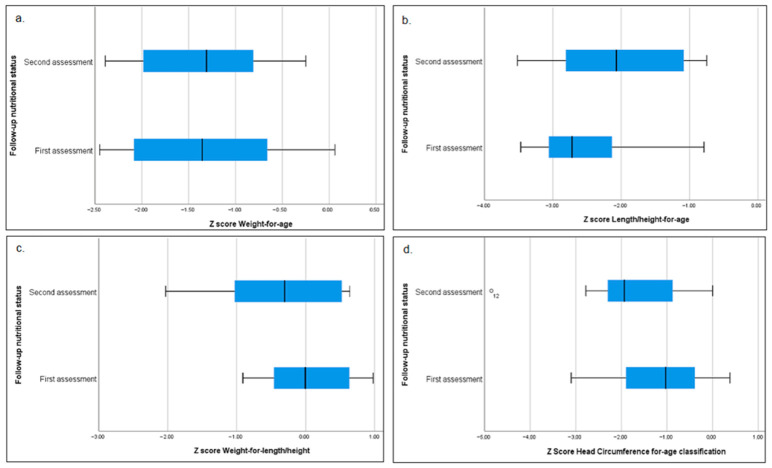
Nutritional monitoring indicators for the longitudinal analysis subgroup of children (**a**). Differences in weight-for-age z-scores between the baseline and follow-up assessments. (**b**). Differences in height/length-for-age z-scores between the baseline and follow-up assessments. (**c**). Differences in weight-for-height/length z-scores between the baseline and follow-up assessments. (**d**). Differences in head circumference-for-age z-scores between the baseline and follow-up assessments.

The follow-up subgroup (n = 24), representing 6.03% of the initial sample, showed a slight increase in the proportion of children with malnutrition or impaired nutritional growth (83.33% to 91.67%); however, this difference should be interpreted with caution given the small subgroup size. A heterogeneous pattern of change was observed (Figure 3):

• 75% of children showed changes in nutritional indicators;
• 41.67% experienced worsening in at least one indicator.

**Figure 4:**
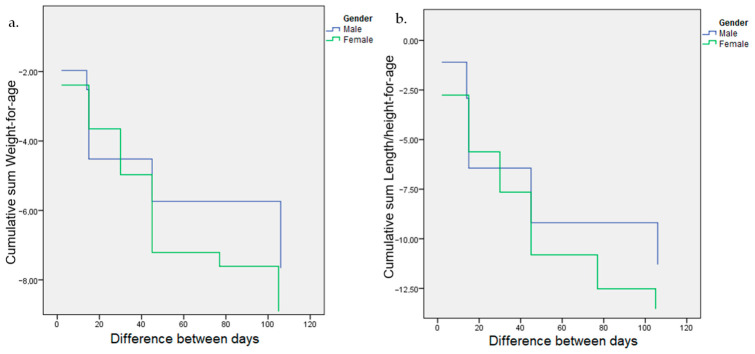
Cumulative anthropometric z-scores (weight-for-age and height/length-for-age) by sex in the longitudinal follow-up subgroup of children under 5 years. The graphs show cumulative z-score changes between baseline and follow-up assessments according to elapsed follow-up time. (**a**). Cumulative weight-for-age z-scores in boys (blue) and girls (green). (**b**). Cumulative height/length-for-age z-scores in boys (blue) and girls (green).

For the follow-up subgroup, paired analyses using the McNemar test showed a statistically significant increase in nutritional risk between visits (83.33% vs. 91.67%; *p* = 0.02). No statistically significant changes were observed in Abnormal head circumference-for-age risk (25% vs. 50%; *p* = 0.61). Graphical analysis (Figure 4), based on cumulative weight-for-age and height-for-age z-scores across visits, suggests a possible decrease in anthropometric indicators within the follow-up subgroup.

### 3.3. Sex and Age Associations

No statistically significant differences were observed in nutritional alterations by sex. However, both underweight (global malnutrition) and stunting (chronic malnutrition) showed statistically significant associations with age. Children aged 2 to 4 years were more likely to present low weight or short stature for age, whereas younger infants were more likely to have normal anthropometric indicators. These findings are consistent with a direct correlation between increasing age and worsening nutritional status (Table 3).

## 4. Discussion

This analysis provides a characterization of the nutritional status of Wayúu children under five years of age in the municipality of Manaure, La Guajira. The findings reveal a critical public health situation, with prevalences of underweight (weight-for-age) and stunting (height-for-age) that are substantially higher than both national estimates and those reported in other high-risk populations, including Indigenous groups in Colombia. Although the prevalence of acute malnutrition (wasting) was lower, it remains approximately twice that reported in the most recent national nutritional survey [31].

Nutritional impairment was significantly associated with age, with a higher prevalence among children older than two years. Within the social, economic, and cultural context of this population, this finding may be related to breastfeeding cessation and the subsequent increased exposure to food insecurity and limited access to resources [32,33,34,35]. The predominance of height-for-age deficit reflects chronic malnutrition characterized by generalized growth impairment, potentially affecting even head circumference. This pattern is insufficiently addressed by current governmental nutritional surveillance strategies, which primarily emphasize weight-for-height indicators and acute malnutrition [36]. In contrast, chronic malnutrition constitutes a distinct and potentially more severe public health challenge.

Recovery from chronic malnutrition or symmetrical growth delay is more difficult to achieve, and its consequences are more profound and long-lasting. These include detrimental effects on neurodevelopment, immune function, and overall functional capacity, with impacts that may persist into adulthood and old age [37,38,39]. Effective intervention requires not only addressing biological and individual-level factors, such as low birth weight, chronic infections (e.g., parasitic diseases), and familial short stature, all of which are present in this population and have been described in similar settings [40,41]—but also tackling the broader structural determinants.

In addition to the previously described socioeconomic factors [32,33,34,35], this population faces severe limitations in access to essential resources, which may exacerbate or perpetuate these biological risks. Evidence from national surveys on multidimensional poverty and nutritional status [25,31,42] indicates that both the municipality of Manaure and the department of La Guajira have some of the highest levels of food insecurity, limited access to basic services (including potable water, sanitation, and electricity), and lower per capita income compared to national averages. These conditions are risk factors that have been associated with chronic malnutrition [32,33,34,35]. Applied to this particular context, they suggest a relationship that, in addition to representing a potential trigger for nutritional alterations, may also operate through reinforcing mechanisms that sustain biological vulnerabilities over time, ultimately contributing to a persistent cycle of poverty–malnutrition–poverty [32,33,34,35,40].

The exploratory longitudinal analysis is limited by the small follow-up subgroup compared with the cross-sectional sample. Despite this limitation, exploratory observations in the follow-up subgroup suggested persistent growth impairment and underweight, along with descriptive changes in anthropometric indicators over time, without clear evidence of recovery. These exploratory findings may suggest descriptive patterns of persistent growth impairment and a potential risk of adverse long-term outcomes; however, they should be interpreted cautiously given the limited sample size. High internal migration between communities also represented a major logistical challenge, affecting research continuity, medical follow-up, and the evaluation of potential interventions.

While the study is primarily descriptive, it highlights the urgent need for sustained and comprehensive interventions in this population. Such strategies should not focus solely on short-term correction of specific anthropometric indicators but rather adopt a holistic and multisectoral approach aimed at addressing both the immediate and underlying determinants of malnutrition. This includes improving access to adequate nutrition, strengthening health services, enhancing ECD programs, and addressing structural inequities that disproportionately affect Indigenous communities.

The main limitations of this study include its observational design, which precludes establishing causal relationships between nutritional outcomes and the factors assessed. In addition, non-probabilistic sampling introduced potential selection bias. Longitudinal follow-up was also limited by geographic barriers and the semi-nomadic lifestyle of the population, which affected cohort retention and the interpretation of longitudinal findings. Furthermore, individual-level socioeconomic and dietary factors were not analyzed, limiting the ability to explore specific associations.

Finally, despite the broad population coverage, these findings reflect the conditions of Indigenous communities within a specific geographic, social, and economic context. Therefore, caution is warranted when generalizing or extrapolating these results, even to other Indigenous or rural populations in Colombia with seemingly similar settings.

## 5. Conclusions

Wayúu children in La Guajira, Colombia, appear to exhibit a shift in nutritional patterns from predominantly acute malnutrition toward chronic growth impairment, highlighting the need to reorient prevention and control strategies for childhood malnutrition. The analysis of malnutrition in this population represents a significant challenge and underscores the need for more comprehensive studies, as the findings suggest potential multifactorial interactions between biological factors and structural socioeconomic determinants that may contribute to the persistence and worsening of nutritional deficits over time.

## Figures and Tables

**Table 1:** Distribution of study participants by sex and Indigenous community.

Indigenous Community	Gender				Total	
	Male		Female			
	*n*	%	*n*	%	*n*	%
Amutka	5	2.3%	5	2.7%	10	2.5%
Cerezal	8	3.7%	11	6.0%	19	4.8%
Cousepa	12	5.6%	5	2.7%	17	4.3%
Gija	7	3.2%	10	5.5%	17	4.3%
Ichieen	2	0.9%	3	1.6%	5	1.3%
Ipalirrakat	13	6.0%	16	8.8%	29	7.3%
Jocolobao	6	2.8%	4	2.2%	10	2.5%
Joumana	8	3.7%	2	1.1%	10	2.5%
Juluwayumana	8	3.7%	4	2.2%	12	3.0%
Kasulepo	6	2.8%	5	2.7%	11	2.8%
Kaurasira	8	3.7%	8	4.4%	16	4.0%
Koushalipa	7	3.2%	7	3.8%	14	3.5%
Mezamana	17	7.9%	6	3.3%	23	5.8%
Monterrey	12	5.6%	11	6.0%	23	5.8%
Palausumana	1	0.5%	2	1.1%	3	0.8%
Paliwoo	4	1.9%	1	0.5%	5	1.3%
Parritchon	5	2.3%	6	3.3%	11	2.8%
Pasito de la Raya	14	6.5%	12	6.6%	26	6.5%
Pausa	9	4.2%	4	2.2%	13	3.3%
Picamana	4	1.9%	9	4.9%	13	3.3%
Polousira	4	1.9%	3	1.6%	7	1.8%
Pondores	5	2.3%	6	3.3%	11	2.8%
Sabana Larga	7	3.2%	0	0.0%	7	1.8%
Samaria (2)	15	6.9%	9	4.9%	24	6.0%
Sinmana	5	2.3%	6	3.3%	11	2.8%
Urraichikat	10	4.6%	8	4.4%	18	4.5%
Yuntamana	14	6.5%	19	10.4%	33	8.3%
Total	216	100.0%	182	100.0%	398	100.0%

**Table 2:** Nutrition indicators among Wayúu Indigenous children under five years from Manaure, La Guajira, Colombia.

Nutritional Indicators	Category	n (p50)	% (ICR)
Nutritional imbalance	Yes	368	92.46
	No	30	7.54
	Total	398	100
Malnutrition or nutritional impairment	Yes	358	89.95
	No	40	10.05
	Total	398	100
Weight-for-age		−1.26	−1.95 a −0.6
Weight-for-age classification	Severely Underweight	21	5.28
	Moderately Underweight	71	17.84
	Underweight Risk	148	37.19
	Normal	147	36.93
	Overweight	11	2.76
	Total	398	100
Length/height-for-age		−2.23	−2.97 a −1.61
Length/height-for-age classification	Severely Stunted	97	24.37
	Moderately Stunted	142	35.68
	Stunted Risk	114	28.64
	Normal	39	9.80
	Tall	6	1.51
	Total	398	100
Weight-for-length/height		0.05	−0.66 a 0.64
Weight-for-length/height classification	Severe Acute Malnutrition	3	0.75
	Moderate Acute Malnutrition	11	2.76
	Malnutrition Risk	44	11.06
	Normal	325	81.66
	Overweight	8	2.01
	Obese	7	1.76
	Total	398	100
Mid-Upper Arm Circumference (cm)		15	14 a 16
Mid-Upper Arm Circumference-for-age		−0.66	−1.29 a 0.08
Mid-upper arm circumference classification	Severe Acute Malnutrition:	1	0.25
	Moderate Acute Malnutrition	7	1.77
	Normal	388	97.98
	Total	396	100
Undernutrition-related mortality risk	Yes	1	0.28
	No	356	99.72
	Total	357	100
Head Circumference for Age		−1.86	−2.72 a −0.89
Head circumference-for-age classification	Microcephaly	185	46.48
	Normal	205	51.51
	Macrocephaly	8	2.01
	Total	398	100

p50: median—IRC: interquartile range.

**Table 3:** Anthropometric indicators by age and sex among Wayúu Indigenous children under five years from Manaure, La Guajira, Colombia.

Nutritional Indicators	Category	Sex n (% *)	Chi-Square Test (χ^2^)	Age in Months p50 (IQR)	Nonparametric Test	Spearman Correlation Nutritional Classification/Age in Months
Weight-for-age classification	Severely Underweight	Male, 198 (3.70%) Female, 13 (7.14%)	0.56	36.82 (23.73–50.53)	<0.01 ++	−0.225 p: <0.01
	Moderately Underweight	Male, 41 (18.98%) Female, 30 (16.48%)		34,85 (22.29–47.30)		
	Underweight Risk	Male, 78 (36.11%) Female, 70 (38.46%)		35.26 (22.57–44.74)		
	Normal	Male, 83 (38.43%) Female, 64 (35.16%)		26.66 (12.00–41.81)		
	Overweight	Male, 6 (2.78%) Female, 5 (2.75%)		12.62 (8.19–35.57)		
Length/height-for-age classification	Severely Stunted	Male, 53 (24.54%) Female, 44 (24.18%)	0.84	31.76 (20.55–43.16)	0.04 ++	−0.099 p: 0.05
	Moderately Stunted	Male, 75 (34.72%) Female, 67 (36.81%)		34.42 (20.18–44.58)		
	Stunted Risk	Male, 64 (29.63%) Female, 50 (27.47%)		36.26 (15.65–44.90)		
	Normal	Male, 22 (10.19%) Female, 17 (9.34%)		25.64 (10.72–40.27)		
	Tall	Male, 2 (0.93%) Female, 4 (2.20%)		8.28 (7.26–14.46)		
Weight-for-length/height classification	Severe Acute Malnutrition	Male, 2 (0.93%) Female, 1 (0.55%)	0.65	14.63 (14.46–55.69)	0.49 ++	−0.010 p: 0.84
	Moderate Acute Malnutrition	Male, 8 (3.70%) Female, 3 (1.65%)		25.21 (15.65–36.82)		
	Malnutrition Risk	Male, 26 (12.04%) Female, 18 (9.89%)		29.93 (20.91–3.47)		
	Normal	Male, 171 (79.17%) Female, 154 (84.62%)		33.23 (17.78–44.74)		
	Overweight	Male, 4 (1.85%) Female, 4 (2.20%)		15.06 (7.12–44.28)		
	Obese	Male, 5 (2.31%) Female, 2 (1.10%)		35.57 (12.62–37.74)		
Head circumference-for-age classification	Microcephaly	Male, 10 (50.0%) Female, 77 (42.3%)	0.23	31.92 (19.26–43.82)	0.34 ++	−0.044 p: 0.38
	Normal	Male, 105 (48.60%) Female, 100 (54,90%)		33.23 (17.62–44.44)		
	Macrocephaly	Male, 3 (1.4%) Female, 5 (2.7%)		15.20 (7.73–39.32)		
Abnormal head circumference-for-age risk	Yes	Male, 111 (51.39%) Female, 82 (45.06%)	0.21	31.82 (18.54–43.82)	0.74 +	N.A.
	No	Male, 105 (48.61%) Female, 100 (54.94%)		33.23 (17.62–44.44)		
Nutritional imbalance	Yes	Male, 198 (91.67%) Female, 170 (93.41%)	0.51	32.58 (18.61–44.36)	0.14 +	N.A.
	No	Male, 18 (8.33%) Female, 12 (6.59%)		28.24 (10.09–41.91)		
DNT/Stunting	Yes	Male, 194 (89.81%) Female, 164 (90.11%)	0.92	33.33 (19.30–44.58)	0.003 +	N.A.
	No	Male, 22 (10.19%) Female, 18 (9.89%)		19.46 (8.14–39.45)		

* % corresponding to the total for each gender separately + U Mann-Whtiney ++ Kruskal–Wallis p50: median IQR: interquartile range.

## Data Availability

The data presented in this study are available upon request from the corresponding author due to legal reasons.

## Abbreviations

The following abbreviations are used in this manuscript:

BMI	Body Mass Index
ECD	Early Childhood Development
FIS	Health Research Fund (*Fondo de Investigación en Salud*)
INS	National Institute of Health (*Instituto Nacional de Salud*)
MUAC	Mid-Upper Arm Circumference
SPSS	Statistical Package for the Social Sciences
WHO	World Health Organization
